# Identification of depression in women during pregnancy and the early postnatal period using the Whooley questions and the Edinburgh Postnatal Depression Scale: protocol for the Born and Bred in Yorkshire: PeriNatal Depression Diagnostic Accuracy (BaBY PaNDA) study

**DOI:** 10.1136/bmjopen-2016-011223

**Published:** 2016-06-13

**Authors:** Elizabeth Littlewood, Shehzad Ali, Pat Ansell, Lisa Dyson, Samantha Gascoyne, Catherine Hewitt, Ada Keding, Rachel Mann, Dean McMillan, Deborah Morgan, Kelly Swan, Bev Waterhouse, Simon Gilbody

**Affiliations:** 1 Department of Health Sciences, Mental Health and Addiction Research Group, University of York, York, UK; 2 Department of Health Sciences, Epidemiology and Cancer Statistics Research Group, University of York, York, UK; 3 York Trials Unit, Department of Health Sciences, University of York, York, UK; 4 Social Policy Research Unit, University of York, York, UK; 5 Hull York Medical School, University of York, York, UK; 6 PNI-UK, Ashbourne, Derbyshire, UK; 7 Children, Women & Families Division, Calderdale and Huddersfield NHS Foundation Trust, Calderdale Royal Hospital, Halifax, UK

**Keywords:** diagnostic accuracy, Whooley questions, perinatal depression, Edinburgh Postnatal Depression Scale, screening

## Abstract

**Introduction:**

Perinatal depression is well recognised as a mental health condition but <50% of cases are identified by healthcare professionals in routine clinical practice. The Edinburgh Postnatal Depression Scale (EPDS) is often used to detect symptoms of postnatal depression in maternity and child services. The National Institute for Health and Care Excellence (NICE) recommends 2 ‘ultra-brief’ case-finding questions (the Whooley questions) to aid identification of depression during the perinatal period, but this recommendation was made in the absence of any validation studies in a perinatal population. Limited research exists on the acceptability of these depression case-finding instruments and the cost-effectiveness of routine screening for perinatal depression.

**Methods and analysis:**

The diagnostic accuracy of the Whooley questions and the EPDS will be determined against a reference standard (the Client Interview Schedule—Revised) during pregnancy (around 20 weeks) and the early postnatal period (around 3–4 months post partum) in a sample of 379 women. Further outcome measures will assess a range of psychological comorbidities, health-related quality of life and resource utilisation. Women will be followed up 12 months postnatally. The sensitivity, specificity and predictive values of the Whooley questions and the EPDS will be calculated against the reference standard at 20 weeks pregnancy and 3–4 months post partum. Acceptability of the depression case-finding instruments to women and healthcare professionals will involve in-depth qualitative interviews. An existing decision analytic model will be adapted to determine the cost-effectiveness of routine screening for perinatal depression.

**Ethics and dissemination:**

This study is considered low risk for participants. Robust protocols will deal with cases where risk of depression, self-harm or suicide is identified. The protocol received favourable ethical opinion from the North East—York Research Ethics Committee (reference: 11/NE/0022). The study findings will be published in peer-reviewed journals and presented at relevant conferences.

**Strengths and limitations of this study**

- This study will fill an important evidence gap regarding the diagnostic utility of depression case-finding instruments for the identification of perinatal depression.
- The study findings will be informed by qualitative interviews conducted with women and healthcare professionals regarding their acceptability of depression case-finding instruments administered during the perinatal period.
- An existing decision analytic model will be updated with current diagnostic accuracy estimates of two depression case-finding instruments, providing an up-to-date estimate of the cost-effectiveness of a perinatal depression screening strategy.
- The study findings will inform policy decisions on the implementation of screening and case-finding strategies for the identification of perinatal depression.
- The study spans four National Health Service (NHS) trusts which may implement differing policies regarding the identification of and referral processes for perinatal depression during the perinatal period.

## Introduction

Depression accounts for the greatest burden of disease of all mental health problems and is estimated to become the second largest cause of global disability by 2020.^1^ It is well recognised that perinatal depression, that is, depression experienced during pregnancy and/or the postnatal period (up to 1 year after birth), is an important category of depression in its own right, with specific guidance provided on the identification and clinical management of the condition.^2^ ^3^

Prevalence rates of perinatal depression vary. Estimates indicate that ∼7.4–20% of women experience depression at some stage during pregnancy^4–6^ with depression during the postnatal period affecting up to 22% of women.^6^ Perinatal depression is associated with a range of adverse outcomes. Evidence suggests an association between depression experienced during pregnancy (prenatal depression) and adverse neonatal outcomes, poor self-reported health, substance abuse and alcohol abuse, and poor usage of antenatal care services.^5^ Postnatal depression has been shown to have a substantial impact on the mother and her partner,^7^ mother–baby interactions,^8^ the family^9^ and on the longer term emotional and cognitive development of the baby,^10^ particularly when depression occurs in the first year of life.^11^

Although perinatal depression is well recognised as a mental health condition, it often goes undetected; with healthcare professionals detecting <50% of cases in routine clinical practice.^12^ The National Service Framework (NSF) for Mental Health states that local protocols should be in place for the management of postnatal depression,^13^ promoting the use of case-finding or screening strategies to aid identification of depression during the perinatal period. This has led to the routine or ad hoc administration of self-report measures such as the Edinburgh Postnatal Depression Scale (EPDS).^14^ Screening or case-finding strategies such as those advocated by the NSF^13^ have since come under scrutiny^15^ ^16^ and have been criticised on a number of factors. Criticisms of the proposed strategies are based on the ethics of mass screening, concerns regarding the psychometric properties of available screening or case-finding instruments (such as variations in diagnostic accuracy estimates and choice of recommended cut-off points for such instruments), the acceptability of such screening or case-finding strategies to patients and healthcare professionals, the paucity of evidence for the cost-effectiveness of screening or case-finding strategies (particularly the costs associated with the management of incorrectly identified cases of perinatal depression), and the absence of any evidence that the process of screening leads to effective management of women with perinatal depression and improved mother and infant outcomes.^16–18^

In 2007, the UK National Institute for Health and Care Excellence (NICE) produced guidelines on antenatal and postnatal mental health.^2^ These set out recommendations for the detection and treatment of mental health problems during pregnancy and the postnatal period. As part of these guidelines, NICE endorsed a case-finding strategy by recommending the use of two ‘ultra-brief’ questions to aid the identification of perinatal depression, with the addition of a ‘help’ question to be asked to those women who answered ‘yes’ to either of the initial case-finding questions (see box 1); these questions are often referred to as the ‘Whooley’ questions.^19^ However, this NICE recommendation was made in the absence of any validation studies of these case-finding (Whooley) questions in a perinatal population. Instead, NICE called for a validation study to be undertaken examining the effectiveness of the Whooley questions against a diagnostic gold standard interview in women during the first postnatal year.^2^ Furthermore, since the commissioning of the current study, NICE have updated their guidelines in which they continue to recommend the use of the Whooley questions during pregnancy and the postnatal period, although they have removed reference to the use of the additional help question.^3^

Box 1 **Whooley questions for identifying perinatal depression recommended by the National Institute for Health and Care Excellence (NICE)^2^**

1. ‘During the past month, have you often been bothered by feeling down, depressed or hopeless?’ (‘Yes’/‘no’).

2. ‘During the past month, have you often been bothered by having little interest or pleasure in doing things?’ (‘Yes’/‘no’).

A third question should be considered if the woman answers ‘yes’ to either of the initial screening questions:

3. ‘Is this something you feel you need or want help with?’ (‘Yes’/‘yes, but not today’/‘no’).

The Born and Bred in Yorkshire—PeriNatal Depression Diagnostic Accuracy (BaBY PaNDA) study therefore aims to close this evidential gap by conducting a validation study of the Whooley questions against a reference standard (the Client Interview Schedule—Revised, CIS-R)^20^ during pregnancy and the postnatal period. Given that the EPDS is the measure most commonly used to detect symptoms of postnatal depression in maternity and child services,^21^ the study will also include a comparative examination of the diagnostic validity of the EPDS. The authors have previously conducted a systematic review commissioned by the National Institute for Health Research Health Technology Assessment Programme (NIHR HTA) of existing methods to identify postnatal depression in primary care.^18^ This revealed a lack of evidence for the validity of the Whooley questions as an identification strategy for postnatal depression. This review has since been updated and found only limited evidence for the use of the Whooley questions as a case-finding strategy for postnatal depression.^22^

The current study builds on pilot work where we have tested the feasibility of longitudinal validation across the perinatal period within the UK National Health Service (NHS) maternity services. This work produced estimates of the diagnostic properties of the Whooley questions in a small but diverse sample of 152 women during pregnancy and the early postnatal period.^23^ The study found that the Whooley questions had a sensitivity of 100% (95% CI 77% to 100%) and a specificity of 68% (95% CI 58% to 76%) during pregnancy, with similar estimates during the early postnatal period (first three postnatal months). Similar positive likelihood ratios were found during pregnancy (3.03) and the early postnatal period (2.73), as was the case for the negative likelihood ratios (0.041 during pregnancy, 0.042 postnatally). The BaBY PaNDA study addresses the need to replicate these results in a larger sample of women representing a wider geographical population spanning different NHS trusts.

If case-finding questions are to be used to aid identification of perinatal depression in routine clinical practice, then it is important that they are acceptable to those women answering the questions and to the healthcare professionals asking the questions. At the time of commissioning the current study, previous research indicated that there were limited studies examining the acceptability to women and healthcare professionals of depression case-finding questions, such as the Whooley questions and the EPDS,^18^ ^24^ although further research has since been conducted in this area.^25^ ^26^ The current validation study will therefore also include an assessment of the acceptability to women and healthcare professionals of such depression case-finding questions and will assess the potential implications for the care pathway for women diagnosed with perinatal depression. This important information will be used alongside the diagnostic estimates of the case-finding questions to inform the implementation of the NICE-endorsed case-finding strategy.

The current study also aims to investigate additional and related aspects of perinatal depression, including the relationship between depression before and after birth and coexisting psychological symptoms. Policy recommendations issued by the UK National Screening Committee (NSC) recognised the need for prospective epidemiological estimates of perinatal depression and psychological comorbidity. Research investigating the natural course of perinatal depression is somewhat limited. Findings from a large longitudinal community sample (the Avon Longitudinal Study of Parents and Children, ALSPAC) suggest higher rates of depressive symptoms in women (as measured by the EPDS) during pregnancy than during the postnatal period (up to 8 months).^27^ Studies which have reported the underdetection of perinatal depression by healthcare professionals are largely drawn from cross-sectional studies of postnatal depression. Further research is needed to determine the degree to which women with prenatal depression continue to be symptomatic in the postnatal period and the proportion of women who are identified as ‘new cases’ in the postnatal period.

Depression is not always experienced in isolation; epidemiological research shows that depression commonly coexists with other common mental health disorders such as general anxiety and somatoform symptoms. Assessments of depression need to recognise and assess for coexisting psychological symptoms to avoid the risk of delivering suboptimal treatment strategies. In line with this, treatment strategies, such as psychosocial interventions, need to consider the full range of comorbid psychological symptoms if they are to be effective. NICE guidance has highlighted the importance of recognising coexisting psychological comorbidity;^28^ however, the issue of psychological comorbidity is not well understood in perinatal mental health research and the current study seeks to address this knowledge gap by assessing women for a range of common mental health disorders.

The current study also seeks to address the concern that screening for perinatal depression is an inefficient way of improving the quality of healthcare for pregnant women and new mothers. The additional health benefit of implementing screening programmes may be limited by factors such as the uptake of the screening programme and the degree to which additional identified cases are well managed and respond to treatment. A major criticism of screening programmes for mental health disorders is that they identify less severe disorders and that these identified cases will remit naturally without the need for any intervention.^29^ To facilitate an understanding of the clinical and economic drivers of the cost-effectiveness of routine screening for postnatal depression, a decision model has been previously developed.^18^ ^30^ A limitation of this model, however, was the limited availability of primary research on the diagnostic utility of depression screening questions and the lack of data on the temporal stability of screening scores and the natural history of screen-positive scores across the perinatal period. The BaBY PaNDA study will provide rich data to help adapt and update this existing decision model for the perinatal period and will enable us to produce robust real-world estimates of the cost-effectiveness of a routine screening and case-finding strategy for perinatal depression.

This prospective validation study will fill important evidence gaps regarding the diagnostic utility, acceptability and cost-effectiveness of depression case-finding instruments. It will inform NICE guidance and UK NSC policy, enabling the NHS to make informed decisions on the implementation of screening and case-finding strategies and to plan services on the basis of rigorous evidence.

### Research objectives

The study will combine epidemiological, psychometric, qualitative and health economic methods to meet a range of clinically important objectives:

1. *Instrument validation*: To determine the diagnostic accuracy of the Whooley depression questions and the EPDS against a reference standard during pregnancy (around 20 weeks gestation) and the early postnatal period (around 3–4 months after birth).
2. *Longitudinal assessment*: To assess the temporal stability of positive and negative screens between pregnancy and the early postnatal period, and to ascertain whether there is an optimal time to screen for perinatal depression.
3. *Assessment of comorbidity*: To investigate the coexistence of depressive symptoms alongside other common mental health problems.
4. *Evaluation of acceptability*: To determine the acceptability of the Whooley depression questions and the EPDS to expectant and new mothers and to healthcare professionals, and the potential implications for the care pathway, during the perinatal period.
5. *Estimates of cost-effectiveness*: To assess the cost-effectiveness of the Whooley depression questions and the EPDS for routine screening for perinatal depression in maternity services.

## Methods and analysis

### Study design

The BaBY PaNDA study is a prospective diagnostic accuracy study and is embedded within the existing Born and Bred in Yorkshire (BaBY) pregnancy and birth cohort study. The BaBY cohort recruits women during pregnancy, along with their partners and babies. Data are collected on maternal and infant health during pregnancy, labour and the neonatal period. Information on the psychological well-being of women and their partners is also obtained during pregnancy and the first postnatal year. The BaBY cohort study has a target population of around 13 500 births per year, with an estimated recruitment rate of >60% of women booked for delivery at each of four hospital sites (York, Hull, Harrogate and Scunthorpe & Goole).

The BaBY PaNDA study will determine the diagnostic accuracy of two depression case-finding instruments (the index tests)—the Whooley questions and the EPDS—against a validated assessment of depression, the CIS-R (the reference standard)^20^ at two stages—once during pregnancy (around 20 weeks gestation) and once during the early postnatal period (around 3–4 months after birth). A 12-month follow-up will also be conducted. Concurrent qualitative and cost-effectiveness evaluations will also be undertaken. The study will take place between April 2013 and June 2016.

### Recruitment

Women will be recruited through the wider BaBY cohort during a 14-month time period.

#### Inclusion criteria

Limited inclusion criteria will be applied to ensure a representative sample of pregnant women are recruited to the study. Eligible women will be identified from the population of women taking part in the wider BaBY cohort study (described above). Pregnant women will be invited to take part in the study if they have consented to take part in the wider BaBY cohort and have consented to be contacted again as part of that consent; are <20 weeks pregnant; are aged 16 years or over; and currently live in an area covered by one of the four hospital research sites.

#### Exclusion criteria

Women will be excluded only if they are non-English speaking. Women with literacy difficulties will not be excluded; in such cases, all study information and questionnaires will be read out to them by the study researchers. Women who are over 24 weeks gestation at the time of receipt of a completed consent form will not be eligible to participate in the study.

#### Recruitment procedure

Recruitment will take place over a 14-month consecutive period across each of the four hospital research sites recruiting to the wider BaBY cohort: York (study coordinating site), Hull, Harrogate and Scunthorpe & Goole. All women who consent to participate in the BaBY cohort and who meet all the BaBY PaNDA inclusion criteria (including having provided consent to be contacted again) will be invited to take part in the study. Eligible women will be sent an information pack at round 15–18 weeks gestation; this will include an invitation letter, a summary information sheet describing the key aspects of the study, a participant information leaflet describing the study in detail, a consent form and a prepaid return envelope. Contact details for the project team will be provided on the information leaflets, should women wish to request further information about the study. Women who wish to take part in the BaBY PaNDA study will be required to complete the consent form and return this to the research team. Women will be contacted by a member of the research team on receipt of a completed consent form to arrange the 20-week assessment. Women who do not return a completed consent form within 2 weeks of receiving the information pack may be contacted by the research team to discuss the study and to provide them with an opportunity to ask further questions about the study.

Information about the BaBY PaNDA study (and the BaBY cohort) will be sent to all general practitioner (GP) practices in the recruiting regions and will be displayed in locations where pregnant women attend as part of their maternity care pathway (eg, antenatal clinics, GP surgeries).

### Index tests and reference standard

The study involves validating two separate index tests against the same reference standard at two separate time points: 20 weeks pregnancy and 3–4 months postbirth. The index tests and reference standard will be administered within the same session by one researcher, with the index tests administered before the reference standard. For cases where it is not possible to administer the index tests and the reference standard in the same session, the reference standard will be administered within 2 weeks of women completing the index tests. The index tests and reference standard will be administered during face-to-face interviews or over the telephone and will be conducted at a time and location according to the woman's preference (eg, antenatal clinic, the woman's home).

#### Index tests

##### Whooley questions

Women will be asked the Whooley questions (see box 1) by a study researcher. A ‘yes’ response to either of questions 1 or 2 will be considered a positive screen for perinatal depression and will require a response to the ‘help’ question (question 3).

The Whooley questions have been previously validated in primary care populations^19^ ^31^ and other clinical populations.^32–34^ Since the design of the BaBY PaNDA study, they have also been validated in small perinatal populations, with sensitivity and specificity estimates in the range of 46–100% and 65–92%, respectively.^23^ ^26^ The Whooley questions were selected as the primary index test as these questions are recommended by NICE to aid identification of depression during the perinatal period^2^ and validation studies for these questions are limited in a perinatal population.

##### Edinburgh Postnatal Depression Scale (EPDS)

Women will be asked to self-complete the EPDS;^14^ this is a 10-item self-report questionnaire measuring depressive symptoms over the past 7 days (eg, ‘I have been so unhappy that I have had difficulty sleeping’, ‘I have felt sad or miserable’). Each item is scored on a four-point Likert scale (0–3), with a total score ranging from 0 to 30. The EPDS has a reported sensitivity of 91% and specificity of 91% when using a cut-off score of ≥13 to detect major depression in the postnatal period.^35^ The EPDS was chosen as one of the index tests as it is a commonly used measure to detect symptoms of postnatal depression in maternity and child services^21^ and is widely used in research in perinatal mental health. It has also been validated for use in pregnancy.^36^

#### Reference standard

##### Clinical interview schedule—revised

Women will be asked to self-complete the computer-based version of the CIS-R.^20^ The CIS-R is a fully structured assessment which assesses 14 areas of symptoms, including depression, anxiety, sleep, fatigue, panic, phobias and compulsions/obsessions, and generates diagnostic categories (including depression severity and diagnosis), according to the International Classification of Diseases (ICD-10) criteria.^37^ Study researchers will be trained in the use and delivery of the CIS-R.

The CIS-R was originally validated in two small primary care samples with good agreement on case definition between clinician and lay interviewers (kappa=0.70 and 0.87), and good correlation between CIS-R scores and clinician-rated severity (kappa=0.77).^20^ It has been used in national psychiatric morbidity surveys^38^ and has also been validated for use over the telephone.^39^ The CIS-R was chosen as the reference standard due to its self-report format.

#### Blinding of outcome results across index tests and reference standard

The index tests and reference standard will be administered in the same session by one researcher. Within a session, the level of potential bias is considered minimal as the EPDS (index test) and CIS-R (reference standard) are both self-report measures completed on paper (EPDS) or on a computer (CIS-R) with only minimal interaction with the researcher. To capture any potential sources of bias, a ‘participant assessment record sheet’ will be completed by researchers following all sessions with women. This will include details of any questions raised by the women during completion of the index tests and reference standard (and any other outcome measures completed as part of the session) and any information provided by the women about their circumstances (past or current).

Blinding of outcome results of the index tests and reference standard will be maintained across the two time points (20 weeks pregnancy and 3–4 months post partum) with different researchers conducting these sessions for each woman, except in those instances where it may be more sensitive for the same researcher to conduct subsequent sessions.

### Outcome measures and data collection

Data collection will occur at three time points during the study:

- Stage 1: prenatal (20 weeks pregnancy);
- Stage 2: postnatal (3–4-month post partum);
- Stage 3: follow-up (12 months post partum).

The main outcome measures will be the two depression case-finding instruments (as the index tests)—the Whooley questions and the EPDS. These instruments will be validated against the CIS-R (as the reference standard). These three measures will be administered at stages 1–3.

Further outcome measures will assess a range of psychological comorbidities with a number of self-report questionnaires administered at stages 1, 2 and 3. These will assess symptoms of depression (Patient Health Questionnaire, PHQ-9^40^); anxiety (Genaralised Anxiety Disorder, GAD-7^41^) and somatic symptom severity (PHQ-15^42^). The CIS-R will also be used to identify other common mental health disorders, including panic disorder, obsessive-compulsive disorder and phobias. Health-related quality of life and health-state utility will be assessed via the Short Form-12 (SF-12)^43^ and European Quality of Life-5 Dimensions (EQ-5D).^44^ Resource utilisation will be captured using a bespoke questionnaire completed at each of the three stages. Acceptability of the depression case-finding instruments to women will be assessed with a self-report acceptability survey originally designed to assess acceptability of the EPDS,^45^ later adapted to include an assessment of the Whooley questions,^23^ and further adapted for use in the BaBY PaNDA study. The acceptability survey will be administered at stages 1 and 2 only. Acceptability of the depression case-finding instruments (to both women and healthcare professionals) will also be determined via in-depth qualitative interviews (see qualitative interviews section for further detail on the assessment of acceptability). Minimal biographic and demographic information will also be obtained at stage 1 only.

Outcome measures will be obtained during face-to-face interviews at stages 1 and 2. At stage 3, and for those women unable to attend a face-to-face interview at stage 2, data will be collected by telephone or a combination of telephone (CIS-R) and post (self-report questionnaires). Face-to-face interviews will be arranged for those women who specifically request this method of data collection at stage 3. Face-to-face interviews will be conducted at a time and place of the women's choosing (eg, antenatal clinic, the women's home). Women are advised via the participant information leaflet and during initial discussions with the study researchers that each session will last ∼30–40 min.

### Sample size

We based the sample size calculation on a previously developed method for diagnostic accuracy studies.^46^ For an expected sensitivity of 95% and a minimal acceptable lower 95% CI of 80% with 0.95 probability, a total number of 50 cases of women with depression in the perinatal period is required. The estimated prevalence of perinatal depression (prenatal and postnatal) is 20%.^6^ Attrition between the prenatal and postnatal stages was estimated at 34%, based on a previous validation study of the Whooley questions in a perinatal population.^23^ Therefore, the sample size needed will be 379 women.

### Qualitative interviews

We will conduct a concurrent mixed-methods qualitative evaluation to determine the acceptability of the Whooley questions and the EPDS to women (both during pregnancy and the first postnatal year) and to healthcare professionals. The interviews will also explore the extent to which they capture appropriate information for effective screening of perinatal depression in routine perinatal care and the potential implications for the care pathway of delivering the depression case-finding instruments in routine care. Interviews will be conducted by a qualitative researcher.

#### Participant interviews

Data collection will include both a quantitative survey (the adapted acceptability survey) to be completed by all women in the study at stages 1 and 2, and in-depth semistructured interviews to be completed with a purposive subsample of 25–30 women. The interview sampling framework will aim for maximum variation on the basis of sociodemographic background, age, parity, positive/negative screens on the Whooley questions and hospital research site. Women will participate in a maximum of three in-depth interviews following completion of the BaBY PaNDA outcome measures at each of stages 1, 2 and 3 to discuss their views of the depression case-finding instruments and their associated experience of the care pathway. Interviews will be conducted on a subsequent and separate occasion to completion of the BaBY PaNDA outcome measures and will be conducted at a time and location according to the woman's preference. Interviews will be guided by the use of a semistructured topic guide based on cognitive interviewing methodology^47^ and open-ended probes. Women will provide their consent to be approached to take part in in-depth interviews at the point of consenting to the BaBY PaNDA study. Women who agree to participate in in-depth interviews will complete a consent form for this aspect of the study.

### Health professional interviews

In-depth semistructured single interviews will be conducted with a purposive sample of six midwives and six health visitors, to include diversity in age, professional grade, experience and hospital site. Interviews will explore health professionals' views and experience of using the depression case-finding instruments as part of routine clinical practice within their NHS trust and their associated training needs, against descriptions of recommended routine practice and policy from health professionals in the respective hospital research site. Health professionals will be provided with an information sheet about the interviews and will be required to complete a consent form prior to conducting the interview.

## Data analysis

### Statistical analysis of diagnostic accuracy data

Two-by-two contingency tables will be used to calculate sensitivity, specificity and predictive values and associated 95% CIs for the Whooley questions and the EPDS against the reference standard (CIS-R) at stage 1 (20 weeks pregnancy) and stage 2 (3–4 months postnatal). Receiver operating characteristics (ROC) curves will be constructed to determine performance characteristics for the Whooley questions and the EPDS at each time point. Indeterminate and/or missing results will be summarised with respect to numbers of women and reasons (if known). The baseline characteristics of women with complete data will be compared with those of women with indeterminate and/or missing data using descriptive statistics. Predictors of non-response will be identified using a logistic regression model if there are sufficient numbers.

Based on the predictive values of the Whooley questions and the EPDS, we will establish which of the two time points (20 weeks pregnancy or 3–4 months postnatal) is better to establish perinatal mental health. The temporal stability of participant responses to the Whooley questions and the EPDS between stages 1 and 2 will be explored using McNemars test. The coexistence of depressive symptoms alongside other common mental health problems at stages 1 and 2 will be summarised descriptively (mean, SD, medium, minimum and maximum, and frequency and percentages at established cut points). Full details will be provided in the statistical analysis plan.

### Qualitative analysis

Interviews will be audio-recorded (with women's consent) and transcribed verbatim. Transcripts will be anonymised to ensure confidentiality. Quantitative data from the acceptability survey will be scored to produce frequency descriptive data on issues relating to acceptability and user preference. Analysis of the qualitative data from the acceptability survey will be subjected to thematic content analysis to include coding of data using constant comparison techniques within the broader context of the existing literature.

The in-depth interviews will be examined holistically using phenomenological research methods on a case-by-case basis to describe women's and health professionals' experience in relation to their own situation and over time.^48–50^ Potential sources of response error for the Whooley questions and the EPDS will be assessed using the cognitive interview approach. The interview data will also be used to further examine the findings from the acceptability survey. The health records of those women participating in in-depth interviews with a positive screen on the Whooley questions at stages 1 or 2 may be examined to triangulate their experience of the depression case-finding instruments and their care pathway.

### Economic analysis

The economic evaluation will be conducted from the NHS and personal social services perspective and will include individual-level quality of life data based on the EQ-5D measure and cost data based on a bespoke resource-use questionnaire. Data recorded on the time taken to fully administer the Whooley questions and the EPDS will also be included. A hypothetical population of pregnant women managed in primary care will be evaluated using a decision analytic model consisting of two parts: (1) an identification model which reflects the diagnostic performance and administration costs of the Whooley questions and the EPDS as perinatal depression identification strategies; and (2) a treatment model which evaluates the health-related costs and outcomes (expressed as quality-adjusted life years, QALYs) that may occur following administration of the depression case-finding instruments. The decision analytic model will be evaluated for true-positive, false-negative, true-negative and false-positive diagnosis groups. Using the diagnostic performance characteristics (sensitivity and specificity values) of the two depression case-finding questionnaires, the impact of true and false identification of perinatal depression and subsequent treatment of perinatal depression on costs and QALYs will be evaluated over the period of the study.

Probabilistic sensitivity analysis using the Monte Carlo simulation method^51^ ^52^ will be undertaken to evaluate uncertainty in parameter estimates in the decision analytic model. To evaluate decision uncertainty, the simulation method will propagate uncertainty in input parameters through the model. Cost-effectiveness plane will be used to present the joint distribution of incremental costs and QALYs. Cost-effectiveness acceptability curves will represent the probability that the Whooley questions are cost-effective compared with the EPDS as a depression case-finding instrument for a range of willingness to pay thresholds that a UK decision-maker may consider.^53^

The decision model will be developed with reference to NICE guidelines for antenatal and postnatal mental health^2^ ^3^ to reflect recommended clinical practice and to ensure that the decision model is realistic and relevant to clinical context.

## Study status

Recruitment of participants is completed. The first participant was enrolled in August 2013. The last participant will complete follow-up (stage 3) in January 2016.

## Ethics and dissemination

### Ethical issues

#### Ethical and safety considerations

As this study does not involve providing any form of intervention, we do not anticipate any major ethical concerns and consider this study low risk for participants. However, we acknowledge that some women may be vulnerable during pregnancy and the postnatal period and may feel anxious about the identification of risk of depressive symptoms. There may also be ethical issues relating to the identification of possible cases of self-harm and/or suicide. Such issues may arise following completion of study outcomes and/or participation in qualitative interviews. Members of the research team have experience of conducting mental health studies and are well placed to deal with such ethical issues. Further, clinical members of the research team will be available to discuss any issues or concerns with researchers and/or the participant, if felt appropriate or requested. We will follow good clinical practice in monitoring risk for self-harm/suicide during researcher encounters with all participants. Robust protocols will be in place to deal with cases where risk of depression, self-harm or suicide is identified or expressed; this may involve contacting the participant's GP where necessary, with the participant's consent.

#### Anticipated risks and benefits

This study is considered low risk for participants. Participants will continue to receive their usual standard of maternity care, and participation in this study will not affect the standard of care they receive from their GP, midwife or health visitor. No treatment will be withheld from participants by their taking part in the study. Information about known risks and possible benefits of taking part in the study will be provided in the participant information sheet. Participants will be informed if new information comes to light which may affect their willingness to participate in the study. The participant information sheet advises potential participants that they may wish to discuss participation in the study with their GP.

#### Obtaining informed consent

Eligible participants will receive an information pack about the study by post. This will contain an invitation letter, a summary information leaflet, a detailed participant information sheet and a consent form. The participant information sheet will provide contact details of the research team should participants wish to request further information about the study or ask any questions before providing their written consent. Researchers will discuss the study with participants and answer any questions during first contact with the participant following receipt of written informed consent.

#### Retention of study documentation

Study data will be stored in accordance with the Department of Health Sciences Data Security Policy at the University of York. Paper records will be stored in secure facilities, and all electronic records will be stored on a password-protected server within the Department of Health Sciences at the University of York. Personal identifiable paper records will be stored in a separate location from anonymised data paper records. All personal information will be destroyed at the end of the study. Anonymised data will be stored for a minimum of 20 years after the final study analysis.

### Dissemination plan

We will publish the findings of this study to include (as a minimum) the diagnostic performance of the Whooley depression questions and the EPDS during pregnancy and the early postnatal period, as well as the findings from the qualitative interviews with participants and health professionals and results of the cost-effectiveness analysis. Findings will be published in peer-reviewed journals and professional journals to ensure accessibility to health researchers and clinicians. Study findings will be published using the Standards for Reporting Diagnostic Accuracy studies (STARD) guidelines.^54^ ^55^ We will present our findings at national conferences on perinatal depression, enabling the effective dissemination of our results to a wide target audience, to include midwives, health visitors, GPs and mental health professionals. We will also issue a press release to ensure coverage of our findings in the wider media. We will produce a short summary of the results for dissemination to all study participants as well as other relevant patient and other interest groups.
